# Comparison of changes in mitochondrial bioenergetics between keratinocytes in human external auditory canal skin and cholesteatomas from normoxia to hypoxia

**DOI:** 10.1038/s41598-017-18536-y

**Published:** 2018-01-09

**Authors:** Cheng-Ping Shih, Jen-Tin Lee, Hang-Kang Chen, Yi-Chun Lin, Hsin-Chien Chen, Yuan-Yung Lin, Chao-Yin Kuo, Yu-Ting Chen, Chih-Hung Wang

**Affiliations:** 1 Department of Otolaryngology-Head and Neck Surgery, Tri-Service General Hospital, National Defense Medical Center, Taipei, Taiwan Republic of China; 2 Department of Otolaryngology, Auditory Medical Center, Cheng Hsin General Hospital, Taipei, Taiwan Republic of China; 3 Graduate Institute of Medical Sciences, National Defense Medical Center, Taipei, Taiwan Republic of China; 4 Graduate Institute of Microbiology and Immunology, National Defense Medical Center, Taipei, Taiwan Republic of China; 5 Taichung Armed Forces General Hospital, Taichung City, Taiwan Republic of China

## Abstract

Cholesteatoma has attracted many studies seeking to uncover its nature and the pathogenesis of related diseases. However, no researchers have explored the mitochondrial bioenergetics of cholesteatoma. The aim of this study was to investigate the energy demand and differential mitochondrial respiration profiles between keratinocytes in external auditory canal (EAC) skin and cholesteatoma samples cultured in normoxic (20% O_2_) and hypoxic (5% O_2_) conditions. Enhanced cellular proliferation of both types of keratinocytes was found in hypoxia compared to normoxia. In 20% O_2_ conditions, cholesteatoma keratinocytes exhibited less mitochondrial mass, lower ATP levels, and significantly lower basal oxygen consumption rate (OCR) and reserve capacity compared to normal skin keratinocytes. In contrast, in hypoxic conditions, cholesteatoma keratinocytes showed markedly higher levels in maximal OCR and reserve capacity, as well as lower proton leak OCRs, compared to normal skin keratinocytes. Hypoxia induced the reverse mitochondrial bioenergy profile from that in normoxia between these two types of keratinocytes, implying that an adaptive change of mitochondrial respiration to oxygen fluctuations may develop in cases of cholesteatoma. Such adaptation in response to hypoxic conditions may play a role in explaining the pathogenesis of acquired cholesteatoma.

## Introduction

Cholesteatoma derives from an abnormal growth of the keratinizing squamous epithelium, which consists of a thin layer of stratified squamous epithelium (matrix) and an adjacent subepithelial connective tissue layer (perimatrix)^1^. The enzymatic activity from matrix components (such as collagenase, plasminogen, and N-acetyl-β-hexosaminidase), the local chronic inflammation–recruited osteoclasts, and the inflammatory mediators concomitant with the disease are responsible for its destruction of the bony external auditory canal (EAC) and ossicular chain^2–7^, resulting in refractory otorrhea and progressive hearing loss. Moreover, vertigo and balance dysfunction can be associated when the labyrinth is involved. Although surgical treatment can cure such disease, a wide range of recidivism rates, from 4 to 61%, has been reported^8–10^; thus, the long-term eradication of cholesteatoma remains a great challenge for otologists.

Previous studies have investigated whether there is an interaction between cholesteatoma and normal skin of the EAC by comparing their gene regulation, biochemical presentation, and cytokine secretion. Cholesteatomas exhibit increased expression of epidermal growth factor receptor (EGFR), transforming growth factor-α (TGF-α), c-jun, and c-myc, accompanied by a significant upregulation of inflammation-related genes and downregulation of several tumor suppressor genes^11–19^. Using whole human genome DNA microarray analysis, the expression profiles of cholesteatomas were demonstrated to mimic those of metastatic tumors and chronically inflamed tissue^14^. The more extensive expression of hyperproliferation-associated cytokeratins exhibited in the cholesteatoma epithelium compared to the EAC epithelium suggested a tight association between cholesteatoma and keratinocyte migration^20,21^. Recent bioinformatics analysis of proteomic data even indicated that a lower grade of differentiation and cancer-like alterations may be involved in the cholesteatoma epithelium^22^. All these published reports strongly suggest that cholesteatoma keratinocytes are distinct from normal skin keratinocytes in many respects.

Multiple theories have been proposed to explain the initiation of cholesteatoma disease, including the following: (1) invagination of the tympanic membrane (retraction pocket), (2) basal cell hyperplasia, (3) epithelial in-growth through perforation (migration theory), and (4) squamous metaplasia of the middle ear epithelium^11–13^. However, the exact etiopathogenesis of acquired cholesteatoma is still unclear. The theory of a combination of invagination and proliferation to explain the retraction pocket in cholesteatoma formation, as described by Sudhoff and Tos^23^, seems to be most applicable. Based on our observations, a retraction pocket of the tympanic membrane is usually secondary to chronic Eustachian tube dysfunction. Once aeration via the Eustachian tube has been interrupted, the middle ear cavity is exposed to decreasing pressure and decreasing oxygenation; in turn, this causes hypoxia and hypercapnia of the middle ear mucosa^24^.

Hypoxia has long been recognized as one of the main promoters in cholesteatoma initiation and progression^25,26^, as hypoxic conditions in the tympanic cavity can result in eardrum retraction pockets developing in the middle ear, followed by cholesteatoma onset and formation. Under hypoxic conditions, hypoxia-inducible factor (HIF-1), one of the main regulators orchestrating the cellular responses to hypoxia, rapidly accumulates and transactivates hundreds of genes, such as matrix metalloproteinases (MMPs), that enhance cholesteatoma deterioration and bone erosion^12^. In addition, the cholesteatoma has been identified as a hypoxic tissue, suggesting that hypoxia may be one of the main promoters in cholesteatoma initiation and progression^25–29^.

To date, there have been no studies on the bioenergetic differences of keratinocytes situated in cholesteatomas and adjacent normal meatal skin, nor have researchers considered the effects of different oxygen tensions on these types of cells. We hypothesized that the mitochondrial bioenergetics characteristics of cholesteatoma keratinocytes (CKs) are different from regular skin keratinocytes of the EAC (EACK) based on the comparative observations described above. The aim of this study was to investigate the differential mitochondrial respiration profiles of these two types of keratinocytes when cultured in normoxia and hypoxia.

## Results

### Cell Growth and Viability under Hypoxic Culture

**Figure 1:**
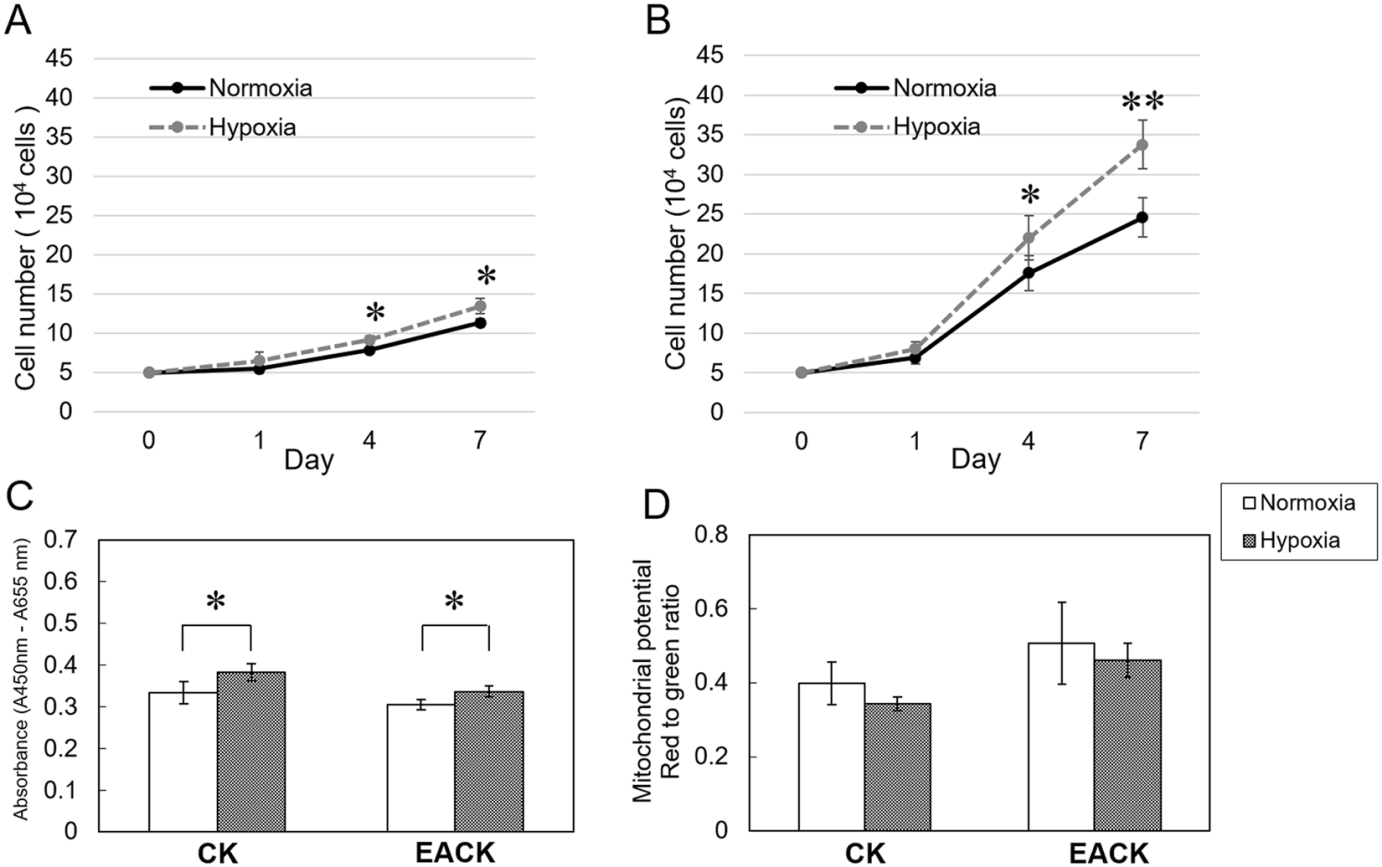
Changes in cell growth and viability between normoxia and hypoxia. (**A**) The cell growth of keratinocytes derived from cholesteatomas and (**B**) the external auditory canal. (**C**) Cell viability of each keratinocyte type cultured in normoxia and 24-h hypoxia was measured using the WST-1 test. (**D**) Concurrent detection of changes in the mitochondrial membrane potential was determined using JC-1 dye. The data shown are pooled from two patients. The results represent the combined data of two separate experiments and are expressed as the mean ± standard error of the mean (SEM), with n = 4 for each bar. *indicates p < 0.05; **indicates p < 0.005; CK = cholesteatoma keratinocyte; EACK = external auditory canal keratinocyte.

The primary culture was successfully established from two patients with paired cholesteatoma and EAC skin biopsy samples. Both epithelial cell types from the cholesteatoma and the EAC skin were found to proliferate well in different oxygen tension culture conditions, as demonstrated in growth studies (Fig. 1). The EACKs showed greater proliferation than the CKs under both normoxic and hypoxic conditions. Compared with normoxic culture conditions on day 4 and day 7, hypoxic culture conditions significantly promoted cell proliferation for both CKs (Fig. 1A) and EACKs (Fig. 1B). WST-1 cell viability assays also demonstrated a similar tendency by showing that hypoxia for 24 h led to increased cell survival compared to normoxia for both the CKs (0.38 ± 0.019 vs. 0.33 ± 0.026, p = 0.025) and EACKs (0.34 ± 0.014 vs. 0.31 ± 0.012, p = 0.014; Fig. 1C).

In testing whether cells retained a stable mitochondrial membrane potential under hypoxic conditions, although the values were relatively low compared to normoxia, the results demonstrated that there was no significant loss of mitochondrial membrane potential in either the CKs or EACKs after 24 h of hypoxia (Fig. 1D). This finding indicated that mild hypoxia obtained by setting a low oxygen (5% O_2_) tension in our experiments may not have interrupted the mitochondrial membrane potentials.

### Changes in Cellular ATP and Lactate Content after 24 h of Growth in Hypoxia

**Figure 2:**
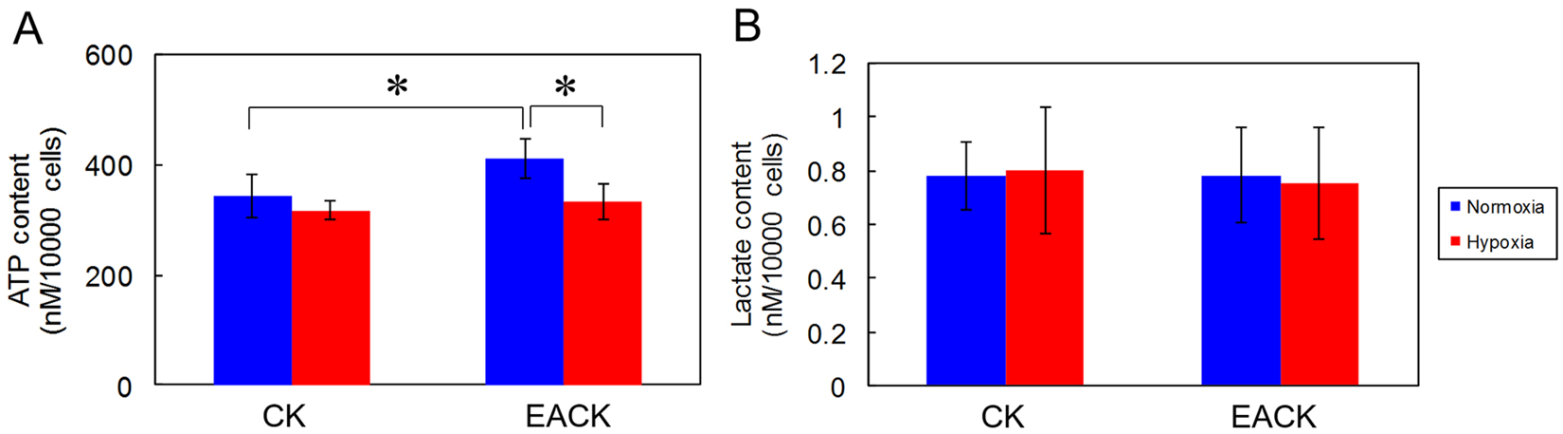
Comparison of the intracellular ATP levels and lactate contents of cholesteatoma keratinocytes and external auditory canal keratinocytes cultured in normoxia and hypoxia for 24 h. (**A**) Both types of keratinocytes after 24-h culture under different oxygen tensions, the cells (2 × 10^5^) were lysed for the determination of cellular ATP levels using an ATP Bioluminescence Assay Kit. (**B**) Culture medium (50 μl) of each sample after 24-h culture under different oxygen tensions were harvested to measure the lactate content using the Lactate Colorimetric Assay Kit. The data shown are pooled from two patients. The results represent the combined data of two separate experiments and are expressed as the mean ± standard error of the mean (SEM), with n = 4 for each bar. *indicates p < 0.05.

We further investigated the effect of hypoxia on cellular respiration between the two cell populations. Figure 2A shows that the ATP level in the CKs was significantly lower than that in the EACKs in normoxia (343.79 ± 39.274 vs. 412.38 ± 36.12, p = 0.042). Following hypoxic exposure for 24 h, there was a decrease in ATP levels for both types of cells, but only the EACKs exhibited a significant difference between normoxic and hypoxic conditions, indicating that hypoxia has a great impact on ATP production in the EACKs.

Concerning the lactate concentration, neither cell population displayed a significant difference in lactate content between 24-h normoxic and hypoxic growth (Fig. 2B). This finding may reflect that, via colorimetric analysis, there is no significantly sudden switch to the anaerobic pathway for either type of cells under hypoxia for 24 h.

### Hypoxic Conditions Induce a Reduction in Mitochondrial Mass in EAC Keratinocytes

**Figure 3:**
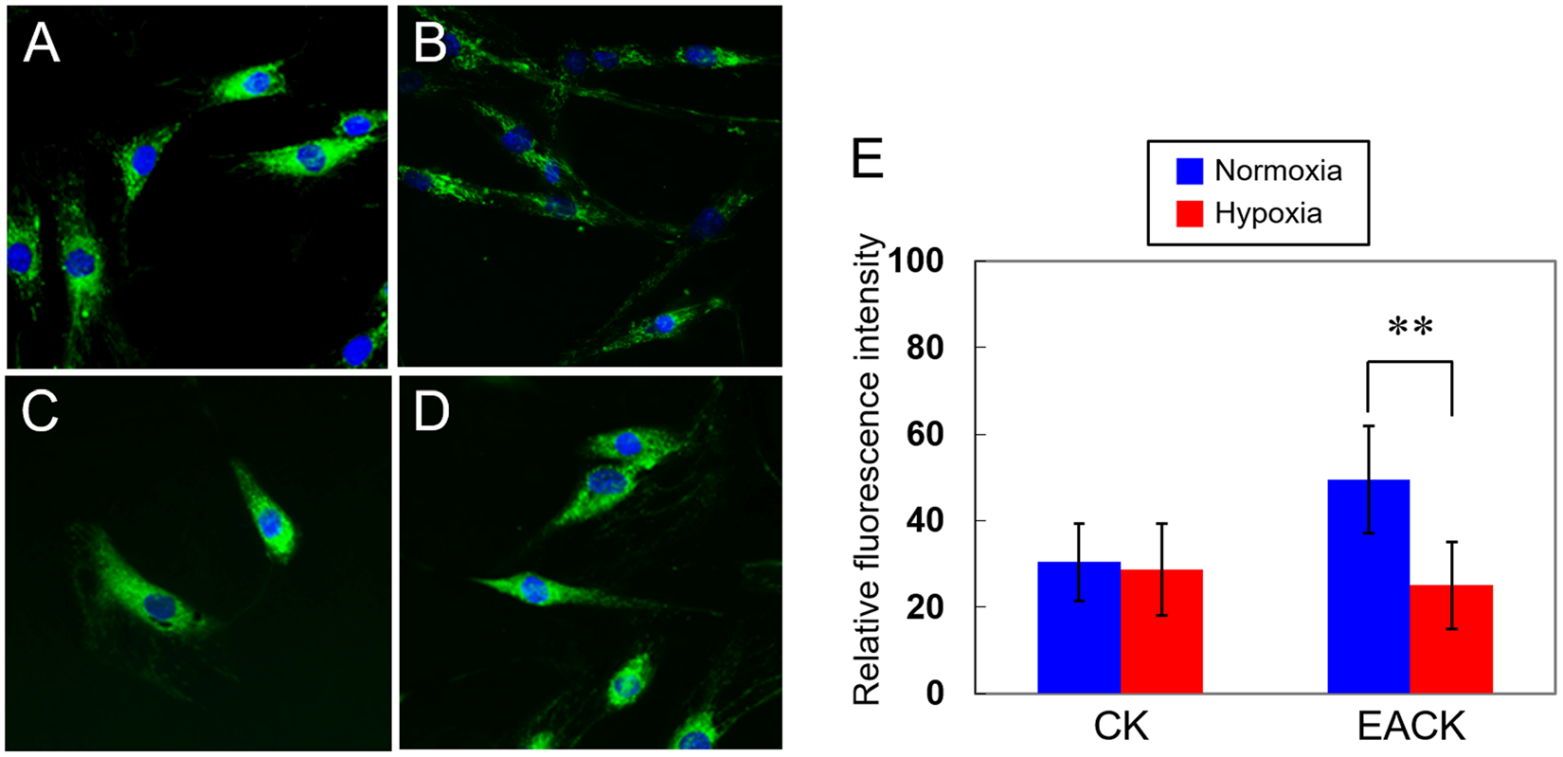
Mitochondrial density differences between the two types of keratinocytes cultured in normoxic and hypoxic conditions. (**A**) Representative images of MitoTracker Green FM staining for the detection of changes in mitochondrial mass in the external auditory canal keratinocytes cultured in normoxia and (**B**) 24 h of hypoxia. (**C**) MitoTracker Green FM staining of cholesteatoma keratinocytes cultured in normoxia and (**D**) 24-h of hypoxia. Nuclei were labeled using Hoechst 33342 (blue). (**E**) Quantification and comparison of mitochondrial densities. The results are expressed as the mean ± standard error of the mean (SEM) with n = 4 for each bar. **indicates p < 0.005.

Subsequently, we examined the mitochondrial density differences between the two types of cells and assessed whether the mitochondrial mass changed after different oxygen tension treatments. In normoxia, a remarkably higher mitochondrial mass was found in the EACKs (Fig. 3A) than the CKs (Fig. 3C). After 24 h of hypoxia, the mitochondrial mass was observed to be decreased in the EACKs (Fig. 3B) but it was maintained in the CKs (Fig. 3D). Simultaneous quantitative analyses of the mitochondrial mass revealed a significant decrease in fluorescence intensity in the EACKs after hypoxia (Fig. 3E), which not only supported the evidence from the MitoTracker Green FM staining images (Fig. 3A–D) but was also consistent with earlier results (Fig. 2A), wherein a significant decrease in ATP levels was observed in EACKs as they transitioned from normoxia to hypoxia. Collectively, these data suggest that keratinocytes derived from the EAC are more sensitive to hypoxic environments than CKs are.

### Hypoxia Results in an Increase in Autophagy in EAC Keratinocytes

**Figure 4:**
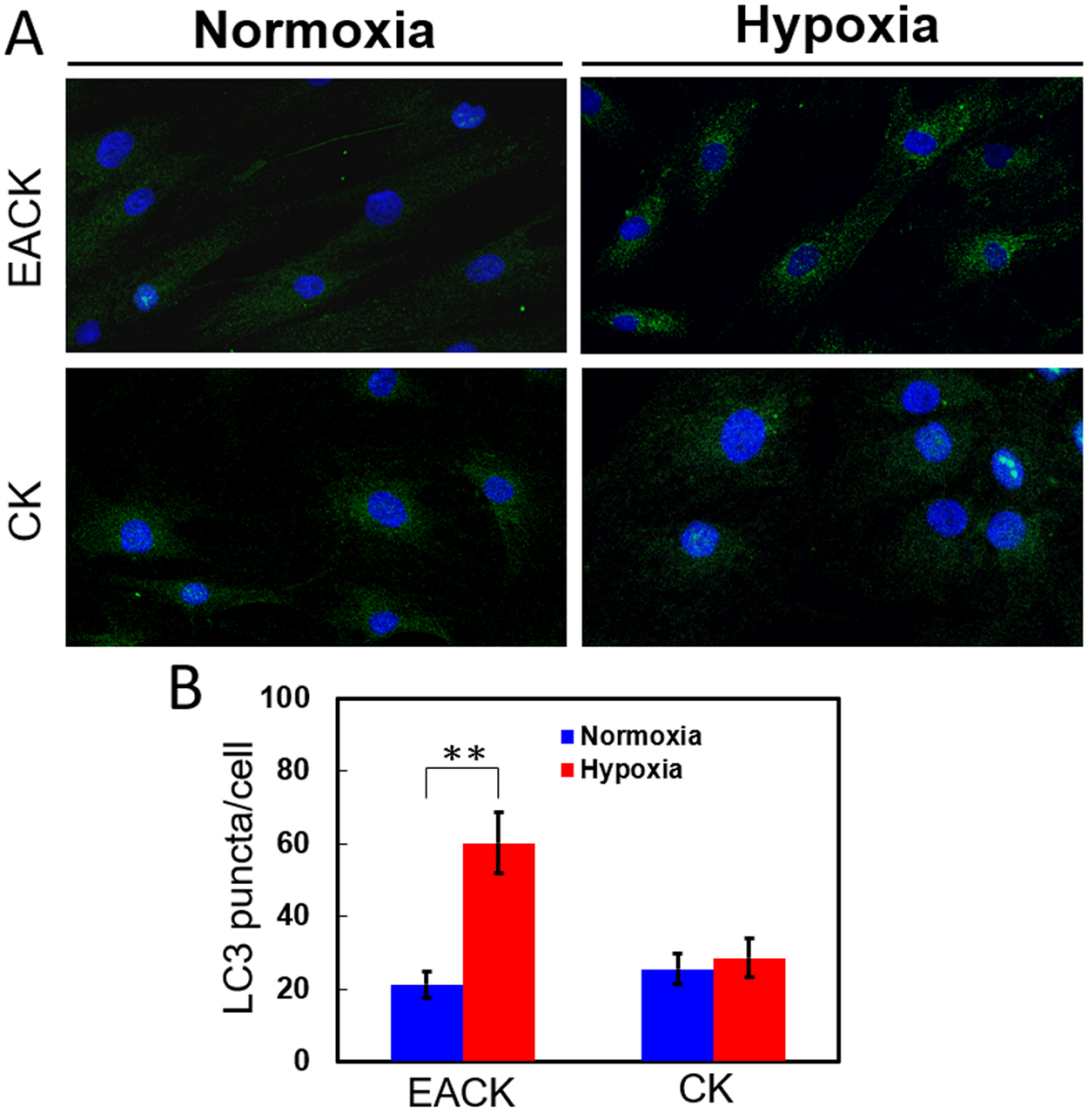
Exposure of EAC keratinocytes to 5% O_2_ for 24 h induced a significant increase in autophagy. (**A**) Representative images of LC3 punctum formation for the detection of autophagy in external auditory canal keratinocytes and cholesteatoma keratinocytes cultured in 20% and 5% O_2_ for 24 h, respectively. (**B**) Quantification and comparison of LC3 punctum formation. The results are expressed as the mean ± standard error of the mean (SEM) with n = 4 for each bar. **indicates p < 0.005.

Autophagy activation has been linked to an alteration in mitochondrial mass^30–33^. To further explore why the mitochondrial mass of EACK was dramatically decreased after 24 h of hypoxic culture, we evaluated the role of autophagy in both types of keratinocytes, especially in response to differential oxygen tension. Interestingly, as shown in Fig. 4, hypoxia induced a marked increase in LC3 punctum formation in the EACKs, whereas no significant autophagy activation, as determined by LC3 staining, was found in the CKs either in the hypoxic or normoxic culture condition. These results not only provide important clues for understanding the effects of differential hypoxia on EACKs and EACK-derived CKs, but also support our hypothesis that CKs transitioned from the EACKs might gradually decrease their mitochondrial mass as they evolve in persistent hypoxic environments. In other words, the transition of EACKs to CKs involves a decrease in mitochondrial mass, which may be associated with the autophagic process of mitochondria.

### The Influence of Hypoxia on Mitochondrial Respiration

**Figure 5:**
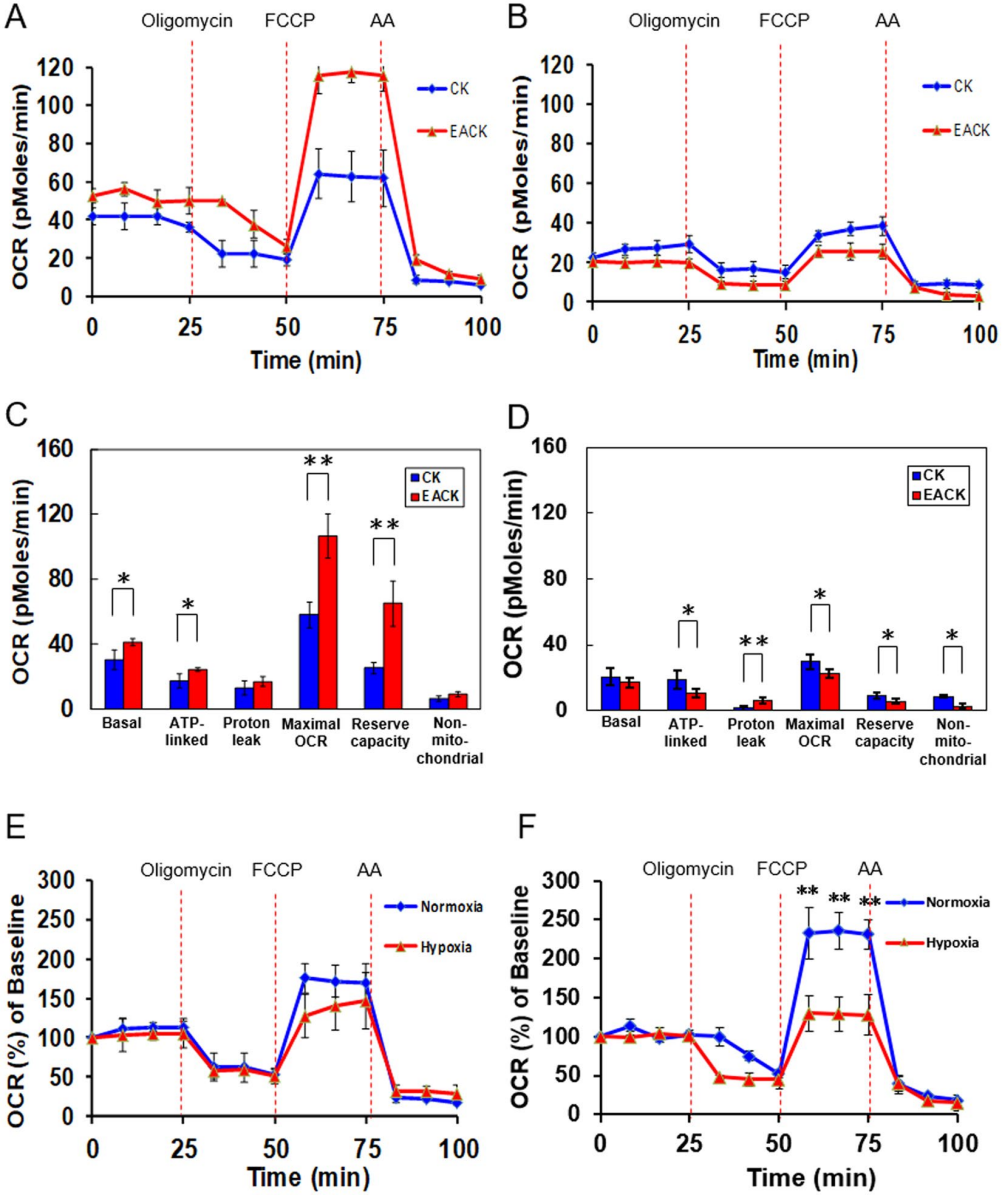
Real-time measurements of the mitochondrial oxygen consumption rates (OCRs) and acute hypoxia effect on the mitochondrial biogenetic profile. (**A**) OCR of cholesteatoma keratinocytes and external auditory canal keratinocytes incubated under normoxia or (**B**) 24 h of hypoxia were plotted under basal conditions and in response to the indicated mitochondrial inhibitors. Each point represents the results of the mean ± standard error of the mean (SEM), with n = 4. (**C**) Quantification of the basal OCR, ATP-linked OCR (basal OCR–oligomycin OCR), proton leak (oligomycin OCR–antimycin A OCR), maximal OCR (FCCP OCR–antimycin A OCR), reserve capacity (FCCP OCR–basal OCR), and nonmitochondrial OCR (antimycin A OCR) of CK and EACK incubated under normoxia or (**D**) 24 h of hypoxia. *indicates p < 0.05; **indicates p < 0.005. (**E**) OCR responses of cholesteatoma keratinocytes cultured in normoxic and hypoxic conditions. (**F**) OCR responses of external auditory canal keratinocytes cultured in normoxic and hypoxic conditions. Data are expressed as a percent relative to the basal OCR before oligomycin, FCCP, or AA injection. All data shown are the mean ± SEM with n = 4. *indicates p < 0.05; **indicates p < 0.005; CK = cholesteatoma keratinocyte; EACK = external auditory canal keratinocyte.

The links between cholesteatoma and EAC keratinocytes and their mitochondrial metabolisms remain unclear. We further characterized the changes in the bioenergetics statuses of the CKs and EACKs at different oxygen tensions using Seahorse’s noninvasive technology. As shown in Fig. 5A and C, the EACK exhibited a higher level of basal OCR in normoxia, implying that keratinocytes derived from cholesteatoma have an altered response to ATP demand. In addition, a significant difference was found in the values of ATP-linked OCR, maximal OCR, and reserve capacity in the EACK, at approximately 1.4-, 1.84-, and 2.57-fold higher than those of the CK, respectively. This finding suggests that CKs are less reliant on mitochondrial respiration than EACKs are; in other words, they consume less oxygen.

In comparing the levels of glycolysis, no significant difference in the ECAR was found between the EACKs and CKs under normoxia (6.6 ± 1.76 mpH/min vs. 4.5 ± 0.53 mpH/min, p = 0.059). This finding suggested that there was no shift to glycolysis in energy production for CKs compared to EACKs, even under a relatively low mitochondrial respiration condition.

In contrast, hypoxic culture resulted in a modestly higher level of basal OCR and much higher levels of maximal OCR and reserve capacity presented in the CKs compared with the EACKs (Fig. 5B and D), indicating that keratinocytes derived from cholesteatomas may have adopted an altered mitochondrial metabolism in response to hypoxic stress or pathologically relevant injury compared to EACKs. In addition, a compensatory metabolic shift involving increasing glycolysis was observed in the EACKs, but not the CKs, after 24 h of growth in hypoxia, as the ECAR value of the EACKs was significantly higher than that of the CKs (9.6 ± 1.65 mpH/min vs. 6.7 ± 0.7 mpH/min, p = 0.018). Unlike the earlier results of the glycolysis assay using the colorimetric analysis to measure lactate, as shown in Fig. 2B, the ECAR represented by the Seahorse XF24 analyzer may be more sensitive and suitable for revealing the glycolysis changes as the earliest events by measuring the proton efflux and metabolic switching of cells in real time instead of as an endpoint. As expected, the basal OCRs of these two cell populations were markedly low in hypoxia compared to normoxia.

As shown in Fig. 5E, when the CK cells were exposed to oligomycin, a natural antibiotic that inhibits F0/F1 ATPase (complex V), the reduced OCR between normoxia and hypoxia showed no significant difference (53% vs. 50.5%). The EACK cells also exhibited a similar percent change in response to different oxygen tensions. This finding suggests that, in both CKs and EACKs subjected to oxygen change, there is no alteration in the ATP demand generated by oxidative phosphorylation (OXPHOS). However, in comparing the maximal rate of oxygen consumption (induced by FCCP) for cells cultured under different oxygen tensions, the CKs showed no significant percentage change, while EACKs behaved quite differently (235% vs. 129.8%, p < 0.001; Fig. 5F). This finding implies that 24 h of hypoxia significantly affected the EACK OXPHOS flux by presenting a less substantial capacity in response to hypoxic stress.

## Discussion

Cholesteatoma has been recognized as an uncontrolled cell growth process. Although various methods of *in vitro* culture systems of human cholesteatoma cells have been introduced, the interpretation of the comparative results of proliferative activity between cholesteatoma and normal skin–derived cells remains controversial. Unlike the observation by Ohnishi, cited by Cheshire *et al*. and Helgaland *et al*.^34,35^, who reported that *in vitro* growth of cholesteatoma cells is faster than that of cells derived from normal EAC skin, our results showed a lower proliferation of cholesteatoma cells than normal skin cells. This finding is in agreement with other studies where cholesteatoma-derived cells demonstrated lower colony-forming efficiency, fewer passages, and a shorter lifespan compared with normal skin–derived cells^35–37^. It is likely that the composition of cells with different growth potentials is more complicated in cholesteatoma than normal skin. For example, a quantitative study of Cheshire *et al*.^35^ showed that the progeny of more highly proliferative cells may only be situated at the neck of the cholesteatoma sac. Lack of perimatrix interaction in the primary culture of cholesteatomas may be responsible for their slower growth. Different methods of preparing or purifying cholesteatoma cells *in vitro* may also explain the conflicting results. In addition, cholesteatoma usually accompanied with repeated infections and inflammation, which may involve several proteolytic enzymes, MMP secretion, and proinflammatory cytokines, thereby interfering with the keratinized epithelial proliferation. As mentioned above, many of the cell proliferation– or infection–related mediators may be a little simplified as reflected in cellular growth curve in an *in vitro* cell culture model.

Notably, hypoxia led to an increase in cell proliferation not only for cholesteatomas but also for normal EAC cells. These findings imply that normal EAC cells have initiated a cellular adaptive program in conditions of limited oxygen supply. Therefore, the hypoxia-related progression of the retraction pocket accompanied by concurrent cellular growth promotion may further support the view that hypoxia plays an essential role in the formation of acquired cholesteatoma. In addition, the positive impact on cell viability of both types of keratinocytes in response to hypoxic stress suggested that oxygen-sensing pathways mediated by HIFs may be involved in both types of keratinocytes to drive this cellular oxygen adaptation^38^.

Previous reports of the *in vitro* culture of human cholesteatoma have mainly focused on investigating the pathogenesis of middle ear cholesteatoma–related growth patterns, differentiation, and involved cytokines^35–37,39–41^. The present study represents the first report of the bioenergetic difference between normal and diseased keratinocytes in human samples. Although hypoxia is suspected to cause cell proliferation via intracellular signaling in both CKs and EACKs, the ATP demand related to oxygen fluctuations is quite different for each type of keratinocyte. Our findings demonstrate that hypoxia decreases the cellular ATP content in EACKs but not in CKs, and this difference may be attributed to the hypoxia-induced autophagic process, as shown in this study. The EACKs, as with most body cells, are sensitive to oxygen change. Thus, exposure to low oxygen tension makes EACKs induce a cell survival response that engages autophagy; by contrast, in CKs, autophagy is significantly less affected by hypoxia, and cells appear to be well adapted to low oxygen tension. In other words, CKs seem to be more tolerant of hypoxic stress than EACKs are. Furthermore, under normal oxygen tension, the lower ATP content and smaller mitochondrial mass shown in CKs suggest that an adaptive mechanism for utilizing oxygen has been developed from EACKs to CKs. Therefore, the significant reductions in basal OCR, ATP-linked OCR, maximal OCR, and reserve capacity shown in CKs compared with EACKs reflect the tight coupling between oxygen consumption and energy demand. In response to hypoxic stress, cells can prevent the increased levels of reactive oxygen species and cell death via an adaptive metabolic response, which is mitochondrial autophagy^31^. Taken together, hypoxia-induced autophagy and changes in mitochondrial mass, as shown in this study, might provide some clues for the underlying transition mechanism between EACKs and EACK-derived CKs.

Additionally, by using the mitochondrial uncoupling agent FCCP to evaluate whether EACKs and CKs change their phenotype in metabolic perturbation, we observed that both types of cells reveal no significant difference in cell proliferation in response to the addition of 1 μM FCCP for 24 h, either in the normoxic or hypoxic culture condition (Supplementary Figure S1). Interestingly, the secretion level of IL-6 resulting from FCCP-induced mitochondrial stress or dysfunction was significantly lower in CKs after 24 h of growth in hypoxia than in normoxia (920.7 ± 51.43 pg/mL vs. 1395.3 ± 108.24 pg/mL, p = 0.0074), implying that a more mitochondrial stress-tolerant mechanism may have evolved in CKs. However, further experiments will be needed to determine the detailed mechanism.

Exposure of both types of cells to hypoxia revealed distinctive results, showing markedly high levels of maximal OCR and reverse capacity accompanied by lower proton leakage in the CKs compared to those in the EACKs. The reserve capacity of CK cells grown under hypoxia was significantly greater than that of EACK cells, implying that cholesteatoma cells may have a greater substantial capacity than normal skin cells in response to hypoxic stress or pathologically relevant injury^27,42^. In addition, proton leakage significantly decreased in the CK at a lower oxygen tension, suggesting that CKs grown under hypoxia may have less mitochondrial membrane damage compared to EACKs. Unlike EACKs, which present an anaerobic glycolysis switch for ATP production during metabolic stress in hypoxic culture condition, CKs present a relative metabolic inflexibility, suggesting that an adaptation for utilizing low oxygen has evolved in CKs. Taken together, our findings suggest that keratinocytes derived from cholesteatomas may have adapted their mitochondrial function to hypoxic environments, thereby ensuring that the mitochondrial respiration activity of the cholesteatoma is intact to fulfill the ATP demand without glycolysis execution in conditions involving such a limited oxygen supply.

Long-term adjustments to hypoxia enable cells or tissues to generate changes in gene expression to improve oxygen and energy supply; this is mainly stimulated by HIFs^43^. Adunka and colleagues demonstrated that cholesteatoma specimens showed more intense staining for HIF-1α and von Hippel–Lindau protein than did normal skin^25^. Interestingly, patients with recurrent cholesteatomas tend to present with higher intensity staining in cholesteatoma tissue than those with primary disease. Because cholesteatoma cells are situated in a long-term hypoxic environment, hypoxia may act as a central mediator in their growth^25,26^. The basal and suprabasal layers of cholesteatomas have been demonstrated to display strong Ki-67 staining for potent proliferation and the highest expression of HIF-1α^44^, indicating that CKs are located in the area of lowest oxygen tension. Our study revealed that hypoxia significantly increased CK proliferation, thereby suggesting that hypoxia is an important facilitating factor in cholesteatoma progression. In this study, we chose 5% oxygen tension, which is closer to the physiological hypoxia condition (around 3%–0.1% oxygen) but not as severe hypoxia or anoxia (<0.1% oxygen), to perform the experiments^45^. All measurements were conducted in an atmospheric environment at the end of each hypoxic and normoxic culture period. The influence of reoxygenation can be an issue and a limitation of our study, although this influence may depend on the severity and duration of hypoxia^46^.

There are two major factors that have contributed to the presence of hypoxia in cholesteatoma. The first is the negative pressure and poor ventilation of the middle ear cavity, where decreased pressure is usually associated with Eustachian tube dysfunction and current or previous otitis media^47,48^. The second factor is that cholesteatoma is often accxpompanied with repeated infection and chronic inflammation, and most importantly, inflammatory disease states are frequently characterized by tissue hypoxia or stabilization of hypoxia-dependent transcription factors, such as HIFs^49^. The association between hypoxia and inflammation represents two sides of the same coin^50^; Eltzschig and Carmeliet emphasized that, in the case of inflamed tissue, hypoxia should not be treated as a bystander but rather as an element that can influence the environment of the tissue, particularly by regulating oxygen-dependent gene expression^51^. As hypoxia can induce inflammation, inflamed lesions can become more hypoxic. Moreover, pathogen-related infection has been proven to increase cellular hypoxia and decrease ATP levels in host cells^52^. Similarly, in this study, we found that the CK exhibited a lower mitochondrial mass, ATP demand, and basal OCR compared to normal keratinocytes. These results can be hypothetically interpreted as an adaptive mechanism to conserve energy in a situation of reduced oxygen availability, inflammation, and infection. Such adaptation in response to oxygen fluctuation—developing into a metabolic bioenergetic difference in cholesteatoma—may explain the role hypoxia plays in the pathogenesis of acquired cholesteatoma.

## Conclusions

In this study, we presented the characteristics of oxygen consumption and energy demand for normal skin and cholesteatoma keratinocytes cultured under different oxygen tensions. Unlike the normal EAC skin keratinocytes, the cholesteatoma cells preserved fewer mitochondria to perform oxidative respiration and exhibited fewer alterations in ATP content and mitochondrial mass in response to oxygen tension changes. Particularly in hypoxic conditions, the CKs exhibited a greater reserved oxygen consumption capacity and less proton leakage in cells compared with normal skin keratinocytes. Such information on the bioenergetic transition between normal skin keratinocytes and cholesteatomas may be helpful for explaining the pathogenesis of acquired cholesteatoma and exploring therapeutic strategies in the future.

## Materials and Methods

### Ethics statement

The study was approved ty the Institutional Review Board of Tri-Service General Hospital (TSGH), National Defense Medical Center, Taipei, Taiwan (Protocol No.: 100-05-045). Written informed consent was obtained from patients prior to surgery for their surgical tissue samples to be used in this research. All procedures were carried out in compliance with the principles of the Declaration of Helsinki (1964).

### Primary cell cultures from surgical specimens

Normal skin tissue of the EAC with a diameter of 5 mm and the cholesteatoma samples were harvested from patients who underwent surgery with a diagnosis of chronic otitis media with cholesteatoma. The samples were immediately placed in sterilized glass tubes containing normal saline for transport to the laboratory. After washing the samples three times with phosphate-buffered saline (PBS), the cholesteatoma and normal skin tissues were finely minced using surgical blades; they were then transferred into a 15-ml centrifuge tube containing 5 ml of 0.05% trypsin and 200 units/ml of penicillin-G for incubation in a shaker at 4 °C. After overnight incubation, 5 ml of Dulbecco’s Modified Eagle Medium/Nutrient Mixture F-12 (DMEM/F12) culture medium supplemented with 10% fetal bovine serum (FBS), 200 units/ml of penicillin-G, and 20 μg/ml of nanomycopulitine was added. The subsequent cell suspension was centrifuged at 1,200 rpm for 5 min. After aspirating and discarding the supernatant, 2 ml of fresh medium was added to suspend the cell pellet. Then, the suspension was placed on collagen-coated 35-mm petri dishes and incubated in humidified atmosphere of 5% CO_2_ and 95% air. The medium was changed three times per week.

After the cellular outgrowth appeared at 7−10 days, the cells were detached by treating them with 0.05% trypsin for 1 min at 37 °C; they were then collected and re-seeded into new flasks containing culture medium. The cells were passaged when they reached 70−80% confluence. Phase contrast microscopy and immunostaining with the epithelial cell marker involucrin and fibroblast marker vimentin were carried out to confirm keratinocyte differentiation. Cells at passages 4−5 were used for all experiments.

### Hypoxia incubation

Hypoxic culture conditions were continuously applied to primary cultured cells in a N_2_/CO_2_ multigas incubator (APM-50D, Astec, Japan) by setting 5% O_2_ low oxygen tension and a 5% CO_2_ atmosphere at 37 °C for the indicated time intervals. For normoxic cultures, cells were cultured at 37 °C with 95% air (20% O_2_) and 5% CO_2_. The medium was changed every 3 days. All measurements were conducted in an atmospheric environment at the end of each hypoxic and normoxic culture period.

### Cell counting and cell viability assay

Cell proliferation was evaluated by counting cell numbers. Cells were seeded in 6-cm dishes at an initial density of 5 × 10^4^ in 2 ml of growth medium, allowed to attach overnight, and then incubated in either normoxic or hypoxic conditions. The medium was changed every 2 days. Resuspended cells were stained with 0.4% trypan blue and measured with a Luna Automated Cell Counter (Logos Biosystems, Gyeonggi-do, South Korea) at the indicated time intervals. For cell viability assay, cells were placed in a flat-bottomed 96-well plate at a density of 2,500 cells per well, allowed to attach overnight, and then incubated in hypoxic or normoxic conditions at 37 °C for 24 h. Medium was replaced with DMEM containing 10% WST-1 (water-soluble tetrazolium salt) assay agent (Roche Applied Science, Mannheim, Germany), and the cells were then incubated for another 4 h. The reaction was catalyzed in active cells by mitochondrial reductase, and the amount of formazan dye could be quantified by measuring the absorbance at 450 nm using a Bio-Rad enzyme-linked immunosorbent assay (ELISA) reader to calculate the optical density (OD) values (A450–A655 nm).

### Measurement of mitochondrial membrane potential

Mitochondrial membrane potential (ΔΨm) was measured using the JC-1 (6-tetrachloro-1,1,3,3-tetraethylbenzimidazocarbocyanine iodide) Assay Kit (Molecular Probes, Invitrogen, Carlsbad, CA, USA) according to the manufacturer’s instructions. Briefly, after incubation in hypoxic and normoxic conditions for 24 h, cells were incubated with 2.5 μg/ml of JC-1 stain for 10 min in the dark and washed twice with PBS. A negative (no JC-1) and positive control (100 μM FCCP (carbonyl cyanide p-trifluoromethoxyphenylhydrazone); Sigma) were also added. Suspensions were washed, pelleted, and resuspended in PBS before analysis with a NucleoCounter^®^ NC-3000 (Chemometec, Denmark). The ratio of red-to-green fluorescence intensity was determined.

### Measurement of cellular ATP

For the determination of cellular ATP levels, an ATP Bioluminescence Assay Kit CLS II (Roche Applied Science) was used following the manufacturer’s protocol. Briefly, cells (2 × 10^5^) were lysed and centrifuged at 10,000 g for 60 s. The supernatant was reacted with luciferase reagent, and the luminescent intensity was measured using a Multi-Mode Reader (Synergy 2, BioTek Instruments, Winooski, VT, USA).

### Mitochondrial mass measurement

Mitochondrial mass was detected using MitoTracker Green FM (Invitrogen). Cells seeded in 35-mm diameter glass bottom dishes (Matsunami Glass Ind., Osaka, Japan) were incubated in hypoxic or normoxic conditions. After 24 h, the medium was removed from the dish, and a prewarmed staining solution containing MitoTracker Green FM (200 nM) and Hoechst 33342 (Cell Signaling Technology, Danvers, MA, USA) was added for the visualization of nuclei. The cells were incubated for 45 min. After three washes with PBS, fresh prewarmed medium was added. Subsequently, the live cells were examined using a Leica DMI6000B inverted microscope (Leica Microsystems, Wetzlar Germany). The images were acquired and analyzed using Image J software for the quantification of fluorescence intensity.

### Autophagy and immunofluorescence microscopy

The cells were grown to 50% confluence on a coverslip. After 24 h of hypoxia, they were washed twice with PBS and fixed with freshly prepared 4% paraformaldehyde at 37 °C for 30 min. Antigen accessibility was increased by treatment with 0.1% saponin for 10 min in BlockPRO blocking buffer (Visual Protein Biotechnology, Taipei, Taiwan). After PBST washes, nonspecific antibody binding was blocked by BlockPRO blocking buffer for 60 min at RT. The cells were incubated with polyclonal primary antibodies to LC3A/B (1:100; Abcam, MA, USA) in an antibody dilution buffer (Dako Co., Carpinteria, California, USA), incubated in a humidified chamber for 1 h at RT, and, after being washed with PBST, stained with a secondary antibody (Donkey anti-rabbit Alexa Fluor 488, 1:500, Molecular Probes) for an additional 60 min. After being washed three times with PBST, the cells were mounted in DAPI Fluoromount-G (SouthernBiotech, Birmingham, AL, USA). Cell images were captured with an LSM 880 Zeiss confocal microscope.

### Measurement of mitochondrial bioenergetics

As in our previous work^27^, an XF24 Analyzer (Seahorse Bioscience, North Billerica, MA, USA) was used to measure the mitochondrial oxygen consumption rate (OCR) and extracellular acidification rate (ECAR) in cholesteatoma keratinocytes (CKs) and EAC keratinocytes (EACKs) under hypoxic and normoxic conditions. Cells were seeded overnight onto an XF24 Cell Culture Microplate (Seahorse Bioscience) at 20,000 cells/well, then incubated under normoxia or hypoxia for 24 h. Before the day of the assay, the cartridge sensor was hydrated overnight with 1 ml of Seahorse Bioscience XF24 Calibration Buffer at 37 °C without CO_2_. On the day of the assay, the growth medium was replaced with serum-free DMEM lacking sodium bicarbonate, and the cells were incubated at 37 °C in a non-CO_2_ incubator for 1 h. OCR and ECAR values were monitored under basal conditions and measured after the injection of oligomycin (1 μM), FCCP (1 μM), and antimycin A (AA, 1 μM) in succession into the well. Data were normalized to the number of cells per well at the end of the culture period and expressed as the OCR in pmol/min and ECAR in mpH/min, and the results were analyzed using the Seahorse XF24 software. Every point represents an average of four different wells. Six parameters of the mitochondrial stress test from the bioenergetics profile were calculated, and these were composed of the basal OCR, ATP-linked OCR, proton leak OCR, maximal OCR, reserve capacity, and nonmitochondrial OCR^28,29^.

### Lactate quantification

To measure the amount of lactate released in the medium, culture medium (50 μl) was harvested from each sample, and lactate content was measured using the Lactate Colorimetric Assay Kit II (BioVision, Milpitas, CA, USA) according to the manufacturer’s instructions. Lactate was oxidized using lactate dehydrogenase to generate a product that interacts with a probe to produce a color (max = 450 nm) measured using a Multi-Mode Reader (Synergy 2, BioTek Instruments).

### Statistical analysis

Statistical analysis was performed using a two-tailed Student’s *t* test. Results are expressed as the mean ± standard error of the mean (SEM). Differences were considered significant at p < 0.05.

## Electronic supplementary material

Supplementary Figure S1
