# Supplementary material for: Comparison of changes in mitochondrial bioenergetics between keratinocytes in human external auditory canal skin and cholesteatomas from normoxia to hypoxia
Source: Sci Rep. 2018 Jan 9;8:125. doi: 10.1038/s41598-017-18536-y (PMC5760563; doi:10.1038/s41598-017-18536-y)
Supplement: Supplementary file 1 — Supplementary Figure S1 [file 41598_2017_18536_MOESM1_ESM.doc]

**Comparison of changes in mitochondrial** **bioenergetics between keratinocytes in human external auditory canal skin and cholesteatomas from normoxia to hypoxia**

Cheng-Ping Shih^1^, Jen-Tin Lee^2^, Hang-Kang Chen^3^, Yi-Chun Lin^3^, Hsin-Chien Chen^1^, Yuan-Yung Lin^1,3^, Chao-Yin Kuo^1^, Yu-Ting Chen^1^ & Chih-Hung Wang^1,3,4,5*^

^1^Department of Otolaryngology-Head and Neck Surgery, Tri-Service General Hospital, National Defense Medical Center, Taipei, Taiwan, Republic of China.

^2^Department of Otolaryngology, Auditory Medical Center, Cheng Hsin General Hospital, Taipei, Taiwan, Republic of China.

^3^Graduate Institute of Medical Sciences, National Defense Medical Center, Taipei, Taiwan, Republic of China.

^4^Graduate Institute of Microbiology and Immunology, National Defense Medical Center, Taipei, Taiwan, Republic of China.

^5^Taichung Armed Forces General Hospital, Taichung City, Taiwan, Republic of China.

**Supplementary information:**


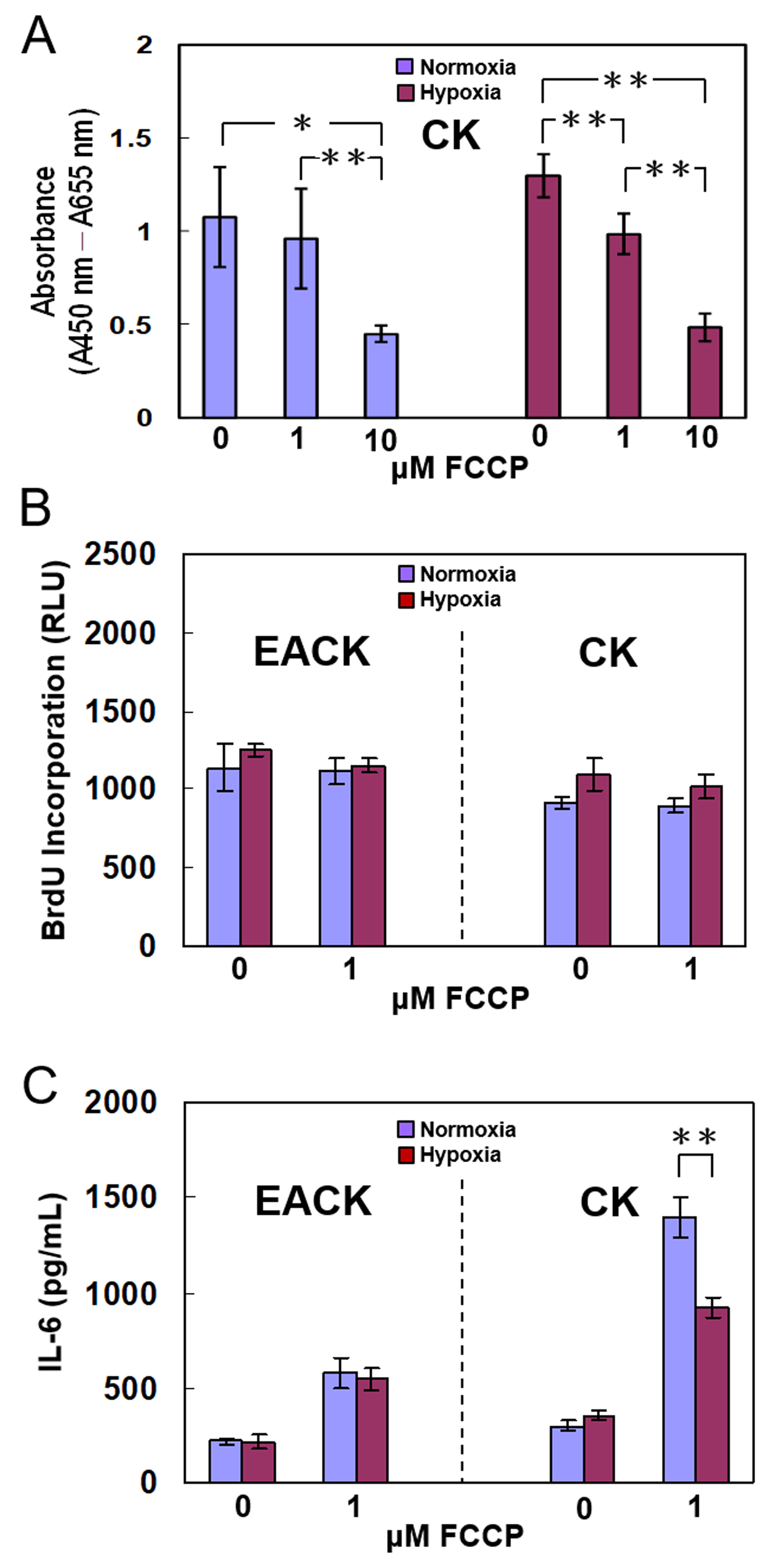


**Supplementary Figure S1.** Effects of the mitochondrial uncoupler (FCCP) on cell viability, proliferation, and IL-6 secretion of external auditory canal keratinocytes and cholesteatoma keratinocytes. **(A)** Cholesteatoma keratinocytes were exposed to various concentrations of FCCP (0, 1, or 10 μM) and cultured in normoxic and hypoxic conditions for 48 h, respectively. Viability was measured using the WST-1 assay. **(B)** Both types of keratinocytes were treated with 0 or 1 μM FCCP and cultured in normoxic and hypoxic conditions for 24 h, respectively. The quantification of cell proliferation was measured using BrdU incorporation. RLU indicates relative light units. **(C)** The levels of IL-6 in the culture supernatants were determined by ELISA. The results are expressed as the mean ± standard error of the mean (SEM), with n = 3 for each bar. * indicates p < 0.05; ** indicates p＜0.005.
